# Disrupting USP5/Cav3.2 interactions protects female mice from mechanical hypersensitivity during peripheral inflammation

**DOI:** 10.1186/s13041-018-0405-4

**Published:** 2018-10-19

**Authors:** Vinicius M. Gadotti, Gerald W. Zamponi

**Affiliations:** Department of Physiology and Pharmacology, Hotchkiss Brain Institute and Alberta Children’s Hospital Research Institute. Cumming School of Medicine, University of Calgary, Calgary, AB Canada

**Keywords:** T-type calcium channel, Ubiquitination, Inflammation, Pain, Sex differences, USP5

## Abstract

Cav3.2 T-type calcium channels are important for the signaling of nociceptive information in the primary afferent pain pathway. During neuropathy and peripheral inflammation, Cav3.2 channels are upregulated due to an increased association with the deubiquitinase USP5. Disrupting these interactions in male mice by the use of cell permeant peptides reverses mechanical and thermal hypersensitivity. Here we explore the effects of interfering with USP5 binding to the channel in female mice with synchronized estrous cycle. We show that intrathecal delivery of a cell-penetrating TAT peptide corresponding to the UBPc domain of USP5 fully reverses mechanical hypersensitivity in mice intraplantarly injected with Complete Freund’s Adjuvant. Hence, the USP5 mediated dysregulation of Cav3.2 channel activity does not exhibit sex differences, and potential therapeutics targeting this interaction should be effective in both male and female subjects.

**Electronic supplementary material:**

The online version of this article (10.1186/s13041-018-0405-4) contains supplementary material, which is available to authorized users.

Cav3.2 T-type calcium channels are important for the propagation and transmission of nociceptive information in the afferent pain pathway [1]. Cav3.2 channel activity is aberrantly upregulated in a number of painful conditions, including visceral inflammation [2], diabetic neuropathy [3] and following nerve injury [4] whereas inhibition of T-type channel activity mediates analgesia [5, 6]. We reported that this enhancement of Cav3.2 channel activity in dorsal root ganglion neurons and spinal cord arises at least in part from an aberrant upregulation of USP5, a deubiquitinating enzyme that binds to the domain III-IV linker region of the Cav3.2 α1 subunit and deubiquitinates the channel [5]. As a result, this channel is protected from degradation, leading to an accumulation of channels in the plasma membrane and consequently larger whole cell currents. This upregulation of USP5 is observed after intraplantar injection of CFA, following peripheral nerve injury, during visceral inflammation, in diabetic mice, and in a model of post-surgical pain [5, 7, 8]. Intrathecal delivery of cell permeant peptides corresponding to either the USP5 interaction site on the channel, or to the Cav3.2 binding site on USP5 reverses mechanical and thermal hypersensitivity associated with these conditions [5, 8, 9]. The studies described above were performed exclusively in male mice. Because there is evidence that certain aspects of pain signaling in the spinal cord exhibit marked sex differences [10, 11], it is important to determine whether disrupting USP5 interactions with Cav3.2 is equally protective in female mice.

To address this issue, we tested the analgesic effects of a Tat-cUBP1-USP5 peptide in female mice. This peptide corresponds to the Cav3.2 interaction site on USP5 and was previously shown by us to be effective in alleviating both mechanical hypersensitivity in CFA injected or nerve-injured male mice [9]. All animal experiments were approved by the Animal care committee of the University of Calgary. Female C57BL/6 J mice were purchased from Jackson laboratories. Their estrous cycle was synchronized and only mice under the proestrus phase were tested. Mice received 20 μl of CFA by intraplantar injection (i.pl.) 2 days prior to behavioral assessment. On the testing day, they were placed individually in plexiglass chambers on top of a grid floor and allowed to habituate for at least 90 min before testing. The Tat-cUBP1-USP5 peptide (10 μg) or vehicle (10 μl) were delivered via intrathecal injections according to methods described previously [12], at a dose that we had previously found effective in male mice [5]. Mice were then tested for mechanical hyperalgesia by assessing mechanical withdrawal threshold via a Dynamic Plantar Aesthesiometer (Ugo Basile, Varese, Italy). Data were then analyzed statistically using two-way analysis of variance.

Figure 1 shows the result of this experiment. Following CFA injection, mechanical withdrawal threshold decreased significantly compared to animals that had received a PBS injection into the hindpaw. Within 15 min of delivery of the Tat-cUBP1-USP5 peptide, there was a near complete reversal of mechanical hypersensitivity that declined over time, but remained statistically different from that observed following intrathecal delivery of vehicle. As in our previous studies with male mice, no behavioral abnormalities were observed following Tat-cUBP1-USP5 treatment. These data indicate that disrupting USP5/Cav3.2 interactions mediate analgesia irrespective of the sex of the animals, and by inference, that the USP5/Cav3.2 pathway is dysregulated in a non-sexually dimorphic way in mice following peripheral inflammation.

**Fig. 1:**
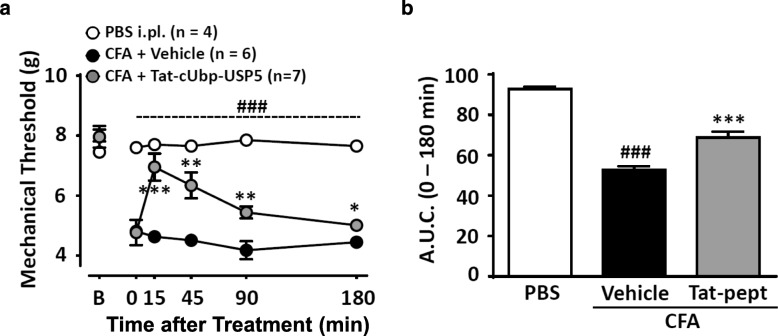
Effects of the Tat-cUBP1-USP5 peptide on inflammatory pain induced by CFA in female mice. **a** Time-dependent effect and (**b**) Area under curve bar representation of Tat-cUBP1-USP5 delivered intrathecally to female mice in the proestrus phase of the estrous cycle. Each point (Panel **a**) represents the mean ± SEM of mechanical withdrawal thresholds of female mice. Bars (Panel **b**) represent mean ± SEM of area under curve for mechanical withdrawal threshold (*n* = 4–7). ### *P* < 0.001 indicates difference between Sham and vehicle control groups and * *P* < 0.05, ** *P* < 0.01, *** *P* < 0.001 denotes significance between vehicle control and Tat-cUBP1-USP5 peptide groups (Two-way ANOVA followed by a Newman-Keuls test)

Closer inspection of the data, however, reveals some differences between male and female mice. First, at the same dose as the one examined here, the maximal effect of the Tat-cUBP1-USP5 peptide in the CFA model was lower in male mice compared to female mice, with males showing a maximal effect of 70–75% reversal of mechanical hypersensitivity. In this context it is noteworthy, however, that full reversal was observed with this dose in a nerve injury model [9]. Second, the kinetics of the action of the tat peptide were different between males and females, such that analgesia peaked after 45 min and declined more slowly in males compared to what we report here. The cellular and molecular basis of these differences is unclear at this point.

Chronic pain is a debilitating condition that is often refractory to treatment, and new avenues for therapeutic intervention are sorely needed. T-type calcium channels have been pursued as a potential pharmacological target for some time. Direct inhibitors of these channels are efficacious in various preclinical pain models, however, there has not been a successful human clinical phase II trial for T-type channel inhibitors [6]. Targeting dysregulation of ion channels is an attractive alternative approach to inhibiting ion flux through the channels, as such an approach that selectively targets channels that are being dysregulated while sparing their normal physiological function is an attractive alternative [13]. This may involve the disruption of protein-protein interactions as such as the one described here, and can potentially be accomplished with small organic molecules rather than cell permeant peptides. Indeed, we have reported that small organic molecules identified in a compound library screen against the USP5/Cav3.2 interaction can show efficacy in various preclinical pain models [7]. The observation that disrupting the regulation of Cav3.2 by USP5 mediates analgesia in both female and male mice further underscores the utility of targeting Cav3.2 channel dysregulation in chronic pain as a possible therapeutic approach.

## Additional file

Additional file 1: Extended Methodology. (DOCX 24 kb)

## Abbreviations

CFA: Complete Freund’s adjuvant
PBS: Phosphate buffer solution
USP5: Ubiquitin specific peptidase 5
