# Supplementary material for: Disrupting USP5/Cav3.2 interactions protects female mice from mechanical hypersensitivity during peripheral inflammation
Source: Mol Brain. 2018 Oct 19;11:60. doi: 10.1186/s13041-018-0405-4 (PMC6194615; doi:10.1186/s13041-018-0405-4)
Supplement: Supplementary file 1 — Extended Methodology. (DOCX 24 kb) [file 13041_2018_405_MOESM1_ESM.docx]

**Materials and methods:**

**Animals**

Female adult mice (C57BL/6J) 9 weeks old were used and purchased from Jackson Laboratories. Only animals under proestrus phase of the estrous cycle were tested following confirmation on testing day. Experiments were approved by the institutional animal care committee. Mice were kept at a maximum number of five per housing cage (30 x 20 x 15 cm) with free to water and food in a room with controlled temperature (23 ± 1°C) and light/dark cycle (lights on at 7am and off at 7pm).

**Persistent inflammatory pain induced by CFA**

To induce thermal hyperalgesia caused by peripheral inﬂammation, animals received 20µl of Complete Freund’s Adjuvant (CFA, Sigma-Aldrich, St. Louis - Missouri, USA) injected i.pl. in the plantar surface of the right hind paw as performed previously. Sham groups received 20µl of phosphate buffered solution (PBS) in the ipsilateral paw. Animals were treated with either a Tat-cUBP1-USP5 peptide (10 µg/i.t.) or vehicle (10µl/i.t.) two days following CFA injection and their mechanical withdrawal threshold was subsequently tested. Experiments were performed using different cohorts of female mice. Baselines were taken before injection of CFA.

Mechanical hyperalgesia was measured using a digital plantar aesthesiometer (DPA, Ugo Basile). Animals were placed individually in a small, enclosed testing arena on top of a grid platform. The device was positioned beneath the testing floor so the filament could be moved and placed accurately under the plantar surface of the ipsilateral paws of each animal. Each paw was tested three times per session.

**Intrathecal injection**

To verify if the Tat-cUBP1-USP5 peptide causes analgesia in female mice, intrathecal (i.t.) injections were performed once to each mouse using volumes of 10μL as routinely performed in our laboratory. Control groups were assessed simultaneously. The Tat-cUBP1-USP5 peptide was dissolved in PBS, whereas vehicle control animals received PBS only. Each mouse was tested only once.

**Estrous cycle synchronization**

Starting 3 days prior to testing, the female mice were transfer to housing cages containing male mice urine. On the testing day, a vaginal smear was collected by lavage of the vagina with 100µl of autoclaved dH_2_O at room temperature using a plastic pipette. The fluid was then expelled on a glass slide and let dry overnight at room temperature. The smears were processed for crystal-violet (0.1%) staining and analyzed by light microscopy (Leica, Germany). The rate of proestrus phase on the 3^rd^ day was 65%.

**Statistical analyses**

Data are presented as mean ± SEM. Statistical analysis was performed by two-way ANOVA followed by Newman-Keuls test, to compare differences between treatments. Differences were considered to reach statistical significance when P < 0.05.
